# 1760. Wastewater-based surveillance for influenza A and RSV and its correlation with clinical disease in Edmonton, Alberta

**DOI:** 10.1093/ofid/ofad500.1591

**Published:** 2023-11-27

**Authors:** Sudha Bhavanam, Judy Qiu, Kristine Du, Nicole Acosta, Nathan Zelyas, Qiaozhi Li, Emily Buss, Kevin Frankowski, Casey R J Hubert, Michael Parkins, Steve Hrudey, Xiao-Li Pang, Bonita Lee

**Affiliations:** University of Alberta, Edmonton, Alberta, Canada; University of Alberta, Edmonton, Alberta, Canada; University of Calgary, Calgary, AB, Canada; University of Calgary, Calgary, AB, Canada; University of Alberta, Edmonton, Alberta, Canada; University of Calgary, Calgary, AB, Canada; Alberta Precision Laboratories, Edmonton, Alberta, Canada; University of Calgary, Calgary, AB, Canada; University of Calgary, Calgary, AB, Canada; University of Calgary, Calgary, AB, Canada; University of Alberta, Edmonton, Alberta, Canada; University of Alberta, Edmonton, Alberta, Canada; University of Alberta, Edmonton, Alberta, Canada

## Abstract

**Background:**

Wastewater (WW)-based surveillance (WBS) of SARS-CoV-2 has been shown to provide a leading indicator to COVID-19 clinical disease including confirmed cases and associated-hospitalizations. To this point, however, longitudinal monitoring of endemic respiratory diseases such as influenza A (IAV) and respiratory syncytial virus (RSV) have minimally been explored. We sought to correlate WW measured endemic virus RNA from municipal WW treatment plants (WWTP) with clinical data to understand WBS performance for endemic respiratory disease in Alberta’s capital city, Edmonton.

**Methods:**

WW was collected thrice weekly from the two WWTP servicing the Edmonton-area (population ∼1.3 million) between Jan’22 & Feb’23. 24-hour composite samples were processed by Centricon ultracentrifugation, and RNA extracted. IAV, and RSV were quantified in duplicate by established RT-qPCR assays. WW SARS-CoV-2 level values adjusted by population served by each WWTP were compared with clinical cases in sewershed-matched areas in Edmonton Health Zone. Daily IAV and RSV case numbers were generated from IAV and RSV specimen testing data extracted from Alberta Precision Laboratory-Public Health Laboratory and FSA of the specimens was used to create the sewershed-matched case data.

**Results:**

A total of 361 wastewater samples from the two WWTPs were collected between January 2022 and February 2023: 83 (30%) tested positive for IAV and 185 (51.2%) for RSV. Over two respiratory viral seasons, 2021/2022 and 2022/2023, IAV peaked in May/June 2022 and again in Nov/Dec 2022. IAV and RSV measured in WW positively correlated with daily confirmed clinical cases within the Edmonton Health Zone (Pearson correlation IAV, *r^2^*=0.61, p< 0.0001; RSV, *r^2^*=0.65, p< 0.0001). Relative to the 2021/2022 season, the WW RSV peak in the 2022/2023 season occurring in November/January was much greater.

Figure 1

**Figure:**
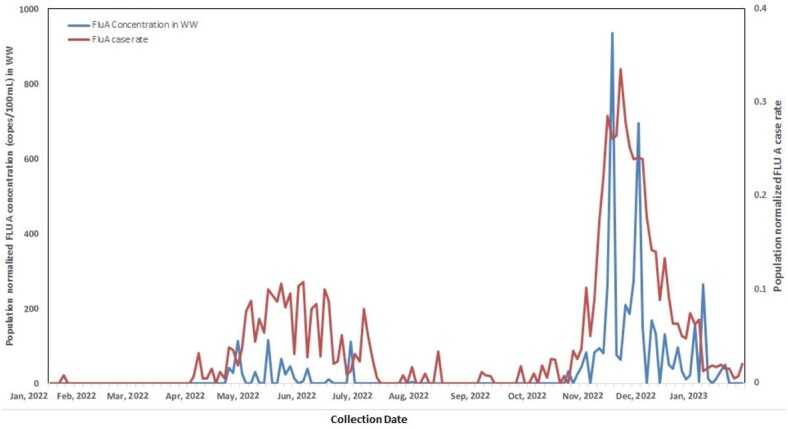

Population normalized Flu A concentrations in wastewater vs. population normalized case rates in the Edmonton Health Zone.

Figure 2

**Figure:**
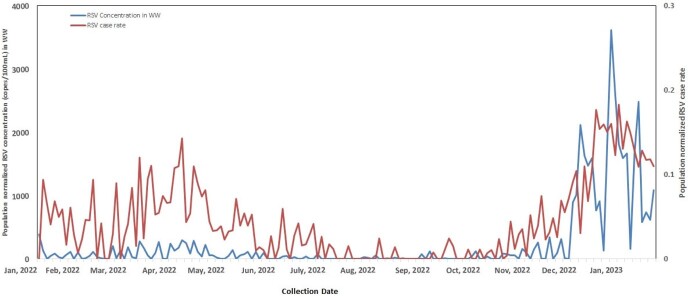

Population normalized RSV concentrations in wastewater vs. population normalized case rates in the Edmonton Health Zone.

**Conclusion:**

WBS of IAV and RSV demonstrated strong correlations with clinically confirmed diseases in sewershed-matched areas in Edmonton, Alberta. WBS enables objective, inclusive and unbiased monitoring of endemic respiratory viral disease activity.

**Disclosures:**

**All Authors**: No reported disclosures

